# Core–Shell Magnetic Nanoparticles

**DOI:** 10.3390/nano13050822

**Published:** 2023-02-23

**Authors:** Alberto López-Ortega

**Affiliations:** 1 Departamento de Ciencias, Universidad Pública de Navarra, E-31006 Pamplona, Spain; lopezortega.alberto@gmail.com; 2 Institute for Advanced Materials and Mathematics (INAMAT2), Universidad Pública de Navarra, E-31006 Pamplona, Spain

The development of novel magnetic core–shell nanoparticles has become increasingly appealing in recent years. This research, in parallel with improvements in the synthesis and fabrication methodologies, has paved the way to obtaining unprecedented multifunctional core–shell structures with unique properties. These types of multiphase nanoparticles can combine the different functionalities of diverse constituents, creating novel and enhanced properties that result in innovative applications.

This Special Issue offers readers a compilation of cutting-edge research regarding the synthesis, development, and characterization of core–shell magnetic architectures, covering a wide spectrum of nanomaterials and serving as a guide for new students of the field as well as established researchers.

In this Special Issue there are research articles that focus on the different types of core–shell magnetic nanoparticles, which have uses ranging from biomedical applications to corrosion stability [1,2]. Moreover, this Special Issue focuses on different synthetic and fabrication methodologies to obtain these types of hybrid structures [1,2,3,4], such as gas phase synthesis [4], colloidal methodologies [2], and mechanochemical preparation [1,4].

In summary, this Special Issue presents several examples of the latest advancements in core–shell magnetic nanoparticles research. We hope that our readers will enjoy these articles and find them useful for their research.
